# Supplementary figures and images for: Glycolaldehyde induces synergistic effects on vascular inflammation in TNF-α-stimulated vascular smooth muscle cells
Source: PLoS One. 2022 Jul 5;17(7):e0270249. doi: 10.1371/journal.pone.0270249 (PMC9255721; doi:10.1371/journal.pone.0270249)

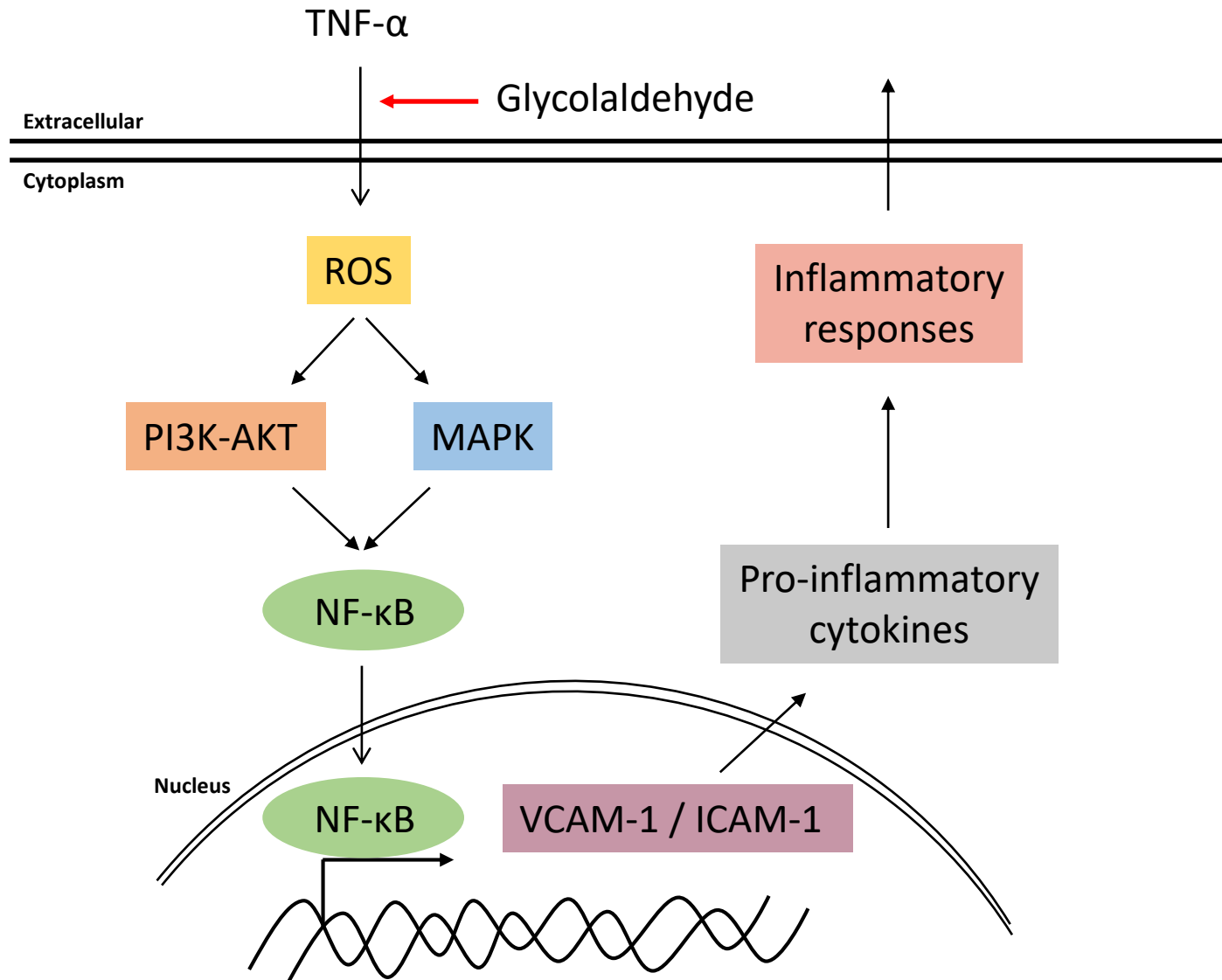

Supplement: S1 Graphical abstract — (PDF) [file pone.0270249.s001.pdf]
