# Supplementary material for: Glycolaldehyde induces synergistic effects on vascular inflammation in TNF-α-stimulated vascular smooth muscle cells
Source: PLoS One. 2022 Jul 5;17(7):e0270249. doi: 10.1371/journal.pone.0270249 (PMC9255721; doi:10.1371/journal.pone.0270249)
Supplement: S1 Raw images — (PDF) [file pone.0270249.s002.pdf]

Western blot (Original images)

Fig. 1(A). Vcam-1

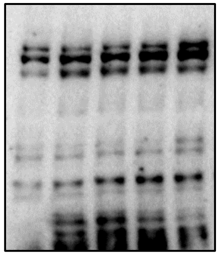

81 kDa ← Vcam-1

Fig. 1(B). Icam-1

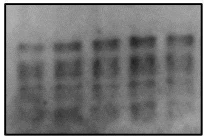

57 kDa ← Icam-1

Fig. 1(E). Vcam-1

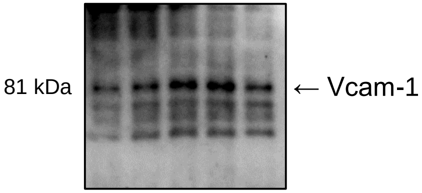

81 kDa ← Vcam-1

Fig. 1(F). Icam-1

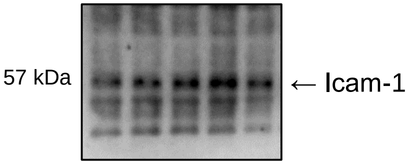

57 kDa ← Icam-1

Fig. 2(A). AGEs

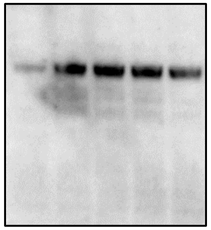

Full kDa ← AGEs

Fig. 3(A). p-p65 (NE)

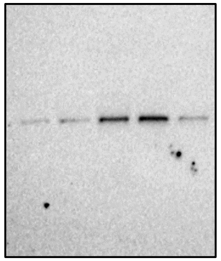

65 kDa ← p65

Fig. 3(A). p-p65 (CE)

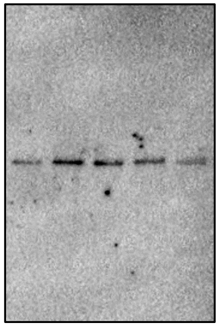

65 kDa ← p-p65

Fig. 3(B). p-IκBα

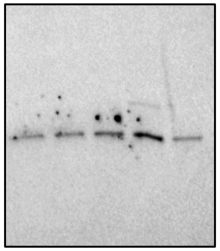

36 kDa ← p-IκBα

Western blot (Original images)

Fig. 3(B). IκBα

36 kDa

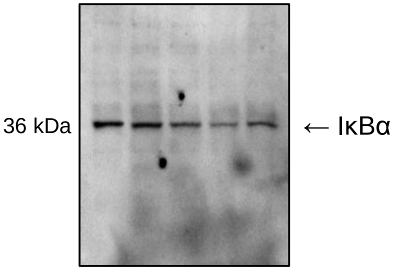

← IκBα

Fig. 4(A). p-ERK

22 kDa

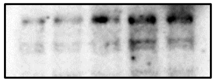

← p-ERK

Fig. 4(A). ERK

43 kDa

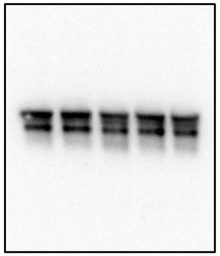

← ERK

Fig. 4(A). p-JNK

48 kDa

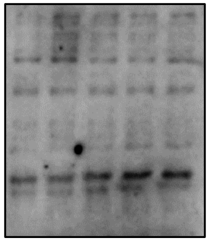

← p-JNK

Fig. 4(A). p-JNK

48 kDa

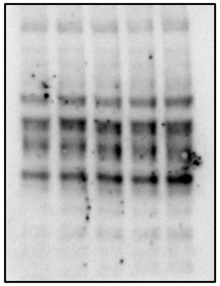

← p-JNK

Fig. 4(A). p-p38

38 kDa

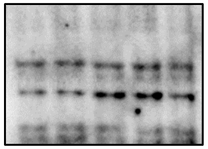

← p-p38

Fig. 4(A). p38

38 kDa

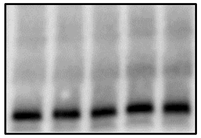

← p38

Fig. 4(B). Vcam-1

81 kDa

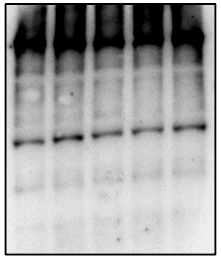

← Vcam-1

Western blot (Original images)

Fig. 4(B). TNF-α

17 kDa

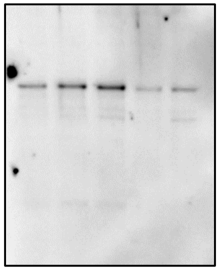

← TNF-α

Fig. 5(C). PI3K

85 kDa

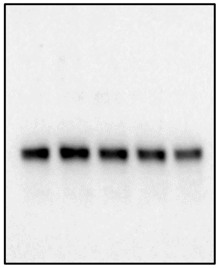

← PI3K

Fig. 6(A). TNF-α

17 kDa

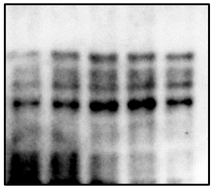

← TNF-α
