# Supplementary material for: Glycolaldehyde induces synergistic effects on vascular inflammation in TNF-α-stimulated vascular smooth muscle cells
Source: PLoS One. 2022 Jul 5;17(7):e0270249. doi: 10.1371/journal.pone.0270249 (PMC9255721; doi:10.1371/journal.pone.0270249)
Supplement: Attachment — Submitted filename: response letter.docx [file pone.0270249.s003.docx]

[September 10, 2021]

Masuko Ushio-Fukai

Editor-in-Chief

*PLOS ONE*

Dear Editor:

I wish to submit our revised manuscript (PONE-D-21-14321) entitled “**Glycolaldehyde induces synergistic effects on vascular inflammation in TNF-α-stimulated vascular smooth muscle cells**” for publication in *PLOS ONE.*

We are grateful to the reviewers for their comments and suggestions, which have helped us to improve our manuscript. We have revised the manuscript based on their comments and have provided our point-by-point responses to each of their comments below.

**Editor’s comment**

1. Especially, purity of nuclear and cytosol fractionation should be shown by including the specific nuclear and cytosolic markers in both fractions. Efffects of GA only should be included. Manuscripts have several mistakes and errors, which should be corrected.

**Response**: We agree with the editor's opinion. Therefore, we separated the nucleus from the cytoplasm and proceeded with the experiment again and used appropriate markers. In addition, we screened the effect of GA alone before the experiment to confirm the effect of GA on atherosclerosis. We confirmed that treatment of GA alone had a significant effect. Therefore, we confirmed the synergistic effect of TNF-α and GA.

2. This study only uses mouse VSMC line, MOVAS-1, which may not reflect intact phenotype. Some key data should be repeated using primary VSMC. In additon, all the averaged graphs should be expressed by dot plots reflecting the individual raw value.

**Response**: Based on the editor's opinion, we experimented with the primary cell HS-VSMC for key results. Also, we replaced all average graphs by expressing them as point plots. (Fig. 1E and F)

**Reviewer 1’s comment**

1. Lee et al found that GA further increased TNFα-induced inflammatory gene expression in mouse VSMC and concluded that GA may contribute atherosclerosis development. This study is not well presented and overstated statements with limited data.

**Response**: We agree with reviewer’s comments. Therefore, we have explained and modified our results within the scope of our study without exaggeration more explicit. (Page 3, Lines 64-65 / Page 10, Lines 232 / Page 11, Lines 246 / Page 12, Lines 280 / Page 13, Lines 285 / Page 15, Lines 352)

2. There are several intermediates for AGE formation such as dicarbonyl compounds, such as methylglyoxal, glyoxal, 3-deoxyglucosone as well as GA. What is the rationale to use GA, not other intermediate, in this study? How GA links with atherosclerosis development?
**Response**: It is really an important factor. In many studies, many studies have been conducted on the occurrence and development of diseases caused by AGEs. According to several studies, various precursors are known to affect blood vessels by producing AGEs. In addition, AGEs play a role in exacerbating diseases due to excessive oxidative stress and inflammatory responses. It was confirmed that GA also induces this response in cells. We determined that various precursors may play a role based on the study of the effects of AGEs. Therefore, various types of precursors were used to evaluate their effects on VSMCs. As a result, it was confirmed that GA had the greatest effect in VSMCs.

Therefore, we conducted this study using GA on atherosclerosis. This study shows for the first time the acute effects of precursors due to vascular oxidative stress and inflammatory responses, which may provide a better understanding of the pathogenesis of atherosclerosis.

3. In introduction, authors described that previous studies reported that deposition of AGE is involved in atherosclerosis via increasing inflammation (Ref 15). Then it is highly expected that AGE precursor, GA involves in inflammation during atherosclerosis. Authors should explain what is new in this study in introduction section.
**Response**: In response to comments from the reviewers, we added a note on the relationship between GA and inflammation in the Introduction section. (Page 4, Lines 76-85 / Page 4, Lines 96 - Page 5, Lines 100)

4. Reference is missing in last paragraph of introduction section (Line 77-86).
**Response**: We have attached references to Lines 77-86 in the Introduction section. (Page 4, Lines 86-93)

5. Give detail about source of GA and aminoguanidine, antibodies (especially AGE, RAGE etc), homogenization buffer etc in method section.
**Response**: We have added sources and information such as chemicals, antibodies and buffers in materials and methods section. (Page 6, Lines 123-124 / Page 6, Lines 130-136 / Page 7, Lines 165- Page 8, Lines 168)

6. As TNF dose mentioned in figures, how authors made 10 nM concentration of TNF-α.
**Response**: We selected a concentration of 10 nM to induce inflammation for the atherosclerotic environment in VSMC, known through many previous studies. (Page 7, Lines 145-146)

7. In Fig 3A, GA increased p-P65 translocation into nucleus but p-P65 in cytosolic fraction also increased. How authors interpret this data?
**Response**: We used beta-actin as a loading control in our existing data. Therefore, it was not possible to know exactly whether the nucleus and cytoplasm were properly separated. We separated the nucleus and cytoplasm again and conducted the experiment again using precise control of the nucleus and cytoplasm, and added accurate results. In the previous study, the phosphorylation form of p65 was increased in the nucleus and cytosol in the TNF-a-induced atherosclerosis model. Our results showed that p65 phosphorylation was increased by GA in the nucleus and cytoplasm. These results were similar to those reported in several previous studies. (Fig. 3A and B)

References

- Kuzhuvelil B. Harikumar, Bokyung Sung, Manoj K. Pandey, Sushovan Guha, Sunil Krishnan and Bharat B. Aggarwal. 2010, 77 (5) 818-827

- Simon Gerhardt, Veronika König, Monika Doll, Tsige Hailemariam-Jahn, Igor Hrgovic, Nadja Zöller, Roland Kaufmann, Stefan Kippenberger and Markus Meissner. 2015, 12-49

8. What is the procedure for nuclear and cytosolic fractionation? Authors need to show successful fractionation using markers.
**Response**: We have added a method for isolating the cytosol and nucleus of cells to the materials and methods section. In addition, cytosol and nuclear markers were identified and used through re-experiment. (Page 8, Lines 176-183 / Fig. 3A)

9. In Fig 4B, recombinant TNF treatment decreased protein expression of TNF by SP600125. What is the mechanism?
Provide replication of each experiment in figure legend.
**Response**: In many previous studies, there have been studies that MAPK is involved in TNF-α-induced VSMCs inflammation and the mechanism of adhesion protein development. Among them, JNK plays a key role in this mechanism. Therefore, we confirmed the effect of MAPK, which plays a key role in the inflammatory response in various immune cells as well as in VSMCs, and determined the effect of each MAPK. In addition, we have provided the replication of each experiment in the figure legend following comments from the reviewers. (Page 25, Lines 578-579 / Page 25, Lines 586-587 / Page 26, Lines 596-598 / Page 26, Lines 606-607 / Page 26, Lines 616-617 / Page 27, Lines 624-625)

10. There are several mistakes and missing reference throughout the manuscript.

**Response**: We reviewed and corrected the mistakes of the manuscript as a whole according to the opinions of the reviewers. We also added missing references. (Page 4, Lines 76-93 / Manuscript)

**Reviewer 2’s comment**

1. In abstract author claims that “This study investigated the effect of GA on the expression of adhesion molecules in the mouse VSMC line, MOVAS-1”. But throughout the figures author did not show any data related to the treatment of GA only. Instead, all data are reflected with the presence of TNF alpha stimulation. TNF alpha is well known pro-inflammatory reagent to induce CAM proteins and MAPK signaling. Therefore, the effect of GA on the inflammation is still in question which is the aim of this study.

**Response**: We understand the comments of the reviewers well. We also initially confirmed the effect of GA alone treatment. However, we confirmed that GA alone had a significant effect. Therefore, we confirmed the synergistic effect of GA by creating an atherosclerotic environment in cells through TNF-α. In addition, we use these data to demonstrate that GA has a synergistic effect on TNF-α-induced atherosclerosis.

2. In addition, the effect of AG inhibitor is not convincing, mainly the Fig 3C, P65 nuclear translocations. Author should provide the good IF images with high resolution to confirm the role of GA on inflammation.
**Response**: We provide high-resolution IF images based on comments from reviewer. Please let us know if you have additional requests for correction of IF images. We will make further corrections. (Fig. 3C)

3. In Fig 1A, TNF-α treatment have no effect on VCAM1 expression which is not acceptable.
**Response**: We confirmed the effect of TNF-α through several screening procedures. Therefore, a result with a clear effect by GA was selected. However, we agree with the reviewer. Thus, we corrected the data for results in which the effect of TNF-α treatment was clearly visible. (Fig 1A)

4. In Fig 3A author should confirm the purity of nuclear and cytosolic fractionation by their respective marker proteins. Actin is not accepted as the loading control for this blot.
**Response**: We confirmed and replaced the cytoplasmic and nuclear markers through re-experiment according to the opinions of the reviewers. (Fig 3A)

5. In Fig 3B, why p-IkBα protein expression is high in basal level while total IkBα protein have no expression in basal.
**Response**: We understand the opinions of our reviewers. We used this band to show the trend of the WB results. We used beta-actin as a loading control in our existing data. Therefore, it was not possible to know exactly whether the nucleus and cytoplasm were properly separated. We separated the nucleus and cytoplasm again and conducted the experiment again using precise control of the nucleus and cytoplasm, and added accurate results. As the reviewer said, we conducted a re-experiment to confirm the exact expression of IkBa and modified it to a more accurate result. (Fig 3B)

6. In Fig 4B, there is a discrepancy in labelling, western blot labelled as TNF-α while the respective quantification in bar graph represents IL-1β.

**Response**: We have corrected the discrepancy between the picture and the cover. (Fig 4B)

We hope that the questions raised by the editors have been adequately addressed and appreciate your prompt attention to this manuscript. We are looking forward to having this paper published in *PLOS ONE*.

Sincerely,

Sang Keun Ha

Korea Food Research Institute

245, Nongsaengmyeong-ro, Iseo-myeon

Wanju-gun, Jeollabuk-do 55365

Republic of Korea

Phone: +82-63-219-9358

E mail: skha@kfri.re.kr
