# Supplementary material for: Glycolaldehyde induces synergistic effects on vascular inflammation in TNF-α-stimulated vascular smooth muscle cells
Source: PLoS One. 2022 Jul 5;17(7):e0270249. doi: 10.1371/journal.pone.0270249 (PMC9255721; doi:10.1371/journal.pone.0270249)
Supplement: Attachment — Submitted filename: response letter.docx [file pone.0270249.s004.docx]

[March 4, 2022]

Jouranl office

*PLOS ONE*

Dear Editor:

I wish to submit our revised manuscript (PONE-D-21-14321) entitled “**Glycolaldehyde induces synergistic effects on vascular inflammation in TNF-α-stimulated vascular smooth muscle cells**” for publication in *PLOS ONE.*

We are grateful to the reviewers for their comments and suggestions, which have helped us to improve our manuscript. We have revised the manuscript based on their comments and have provided our point-by-point responses to each of their comments below.

**Reviewer 1’s comment**

1. For subcellular fraction (cytosolic and nuclear) assay, the procedure is not well described. What is meaning of buffer A, buffer B. Which kit used for this experiment. In figure 3A, what is the expression level of tubulin in NE and what is the level of Lamin A in CE. This will give the purity of fractions.

**Response**: We added a description of the separation of the nuclear and cytosol. (Page 8, Lines 176-186) In addition, experiments were conducted to confirm the separation of the nucleus and cytoplasm.

2. What software used for statistical analysis. Which test used for ANOVA.

**Response**: We added the method used for statistical analysis. (Page 9, Lines 206-210)

3. In response to replication of experiments, Authors mentioned “quintuplicates” in figure legend. This will confuse the readers, Authors should follow the journal format (such as N=5).

**Response**: We modified it to fit the journal format, as mentioned by reviewers. (Page 25, Lines 594; Page 26, Lines 602, 613; Page 27, Lines 622, 632, 640)

4. Several representative blots are not good (Fig 2B; Fig 4A; Fig 6B), Replace with representative blots.

**Response**: We modified the quality of the bands mentioned. (Fig 2B, Fig 4A, Fig 4B, Fig 5C, Fig 6B)

**Reviewer 2’s comment**

1. In the section of method and materials “cytosol and nuclear extract preparation” protocol needs detailed information about the kit (like company name and catalog number) or what is the composition of “buffer A” and “buffer B”.

**Response**: We added information about the kit and composition to the buffer. (Page 8, Lines 176-186)

2. Fig 2B immunoblot images are not acceptable for publication. Image’s qualities need to be improved.

**Response**: We modified the quality of the bands mentioned. (Fig 2B)

3. In Fig 4B immunoblot, 3rd lane and 5th lane labelling are same, but the TNFa and VCAM1 expression in 3rd and 5th lane are completely different. Author should re-check the labelling and the quantification graph as well.

**Response**: We checked the bands again and corrected the quantitative graph. (Fig 4B)

We hope that the questions raised by the editors have been adequately addressed and appreciate your prompt attention to this manuscript. We are looking forward to having this paper published in *PLOS ONE*.

Sincerely,

Sang Keun Ha

Korea Food Research Institute

245, Nongsaengmyeong-ro, Iseo-myeon

Wanju-gun, Jeollabuk-do 55365

Republic of Korea

Phone: +82-63-219-9358

E mail: skha@kfri.re.kr
